# Robust, motion-free optical characterization of samples using actively-tunable Twyman–Green interferometry

**DOI:** 10.1038/s41598-023-32791-2

**Published:** 2023-04-07

**Authors:** Arjent Imeri, Syed Azer Reza

**Affiliations:** Department of Physics and Optical Engineering, Rose-Hulman Institute of Technology, Terre Haute, IN 47803 USA

**Keywords:** Engineering, Optics and photonics

## Abstract

Optical interferometry-based techniques are ubiquitous in various measurement, imaging, calibration, metrological, and astronomical applications. Repeatability, simplicity, and reliability of measurements have ensured that interferometry in its various forms remains popular—and in fact continues to grow—in almost every branch of measurement science. In this paper, we propose a novel actively-controlled optical interferometer in the Twyman–Green configuration. The active beam control within the interferometer is a result of using an actively-controlled tunable focus lens in the sample arm of the interferometer. This innovation allows us to characterize transparent samples cut in the cubical geometry without the need for bulk mechanical motion within the interferometer. Unlike thickness/refractive index measurements with conventional Twyman–Green interferometers, the actively-tunable interferometer enables bulk-motion free thickness or refractive index sample measurements. With experimental demonstrations, we show excellent results for various samples that we characterized. The elimination of bulk motion from the measurement process promises to enable miniaturization of actively-tunable Twyman–Green interferometers for various applications.

## Introduction

Optical interferometry remains the technique of choice in various branches of scientific research and applications^1^. The use of optical interferometry ranges from applications in optical imaging, optical metrology, optical ranging and sensing, to applications in astronomy and astrophysics, such as its use in gravitational wave detection^2^ as well as in radio astronomy^3^. There are various reasons for the ubiquitous acceptance and applicability of optical interferometry in measurement systems. These include the high measurement sensitivity and repeatability that optical interferometry-based techniques are able to deliver.

Despite a similar underlying operation principle, various interferometric configurations offer different modes of measurements. For example, interferometers can employ different light sources which may vary in coherence and spectral properties. Michelson, Mach-Zehnder and other similar interferometers that use quasi-monochromatic sources often rely on counting fringe shifts to measure and track gradual changes in the quantity of interest. For example, these interferometric configurations can be used to measure changes the pressure or temperature inside chambers^4,5^ via fringe counting. On the other hand, techniques such as optical coherence tomography^6^ use a low coherence source to scan one sample depth at a time. Likewise, different interferometers can differ in measurement modalities in terms of making either spatial, temporal or spatio-temporal measurements. Moreover, interferometers can also differ in terms of the type of quantities which they can measure as well as their relative resolutions and measurement dynamic range.

Other than measuring gradual changes to the differential optical path length between the sample and reference arms, interferometers can also be utilized for measuring abrupt changes to the differential path length. This is the case when an optical sample is inserted in the sample arm of an interferometer such as the Mach-Zehnder or the Michelson types. In such cases, fringe counting is not possible due to the non-gradual change in the path length and enabling sample characterization for interferometers which rely on gradual changes requires introducing some gradual path length changes after sample insertion such as employing sample rotation and counting fringes as the sample rotates^7^.

A conventional Twyman–Green (TG) interferometer^8^, offers a solution where a sample can be characterized^9–12^ despite the abrupt chance in the optical path length of the sample arm upon sample insertion to an initially ’balanced’ interferometer configuration. The largest (zero-order) interference fringes are recorded at the detector when the TG interferometer is in the balanced state. The TG interferometer is able to measure abrupt changes to path length by measuring the change which is required to re-balance the interferometer after sample insertion. Mostly, re-balancing the TG interferometer requires translating the sample arm mirror M until the interferometer re-balances. The motion required to achieve re-balancing is the used to determine sample properties such as its thickness or refractive index.

Evidently, spatial interferometric techniques for sample characterization mostly require some type of bulk optical motion (rotational, translational etc.) to enable sample characterization. Avoiding bulk mechanical motion requires either making temporal time-of-flight measurements^13^ or resorting to heterodyne interferometric techniques^14,15^ where changes in the low-frequency optical beatnotes are tracked and analyzed (spatially or temporally) to estimate changes in optical path length. These setups are often more expensive in comparison to basic interferometers and require substantial additional hardware or upgrades to existing hardware.

Tunable focus lenses (TFLs) are electronically reconfigurable and their focal length is easily tuned with a change to the applied input current/voltage signal to the lens. While TFLs are a part of various imaging and metrological systems^16–23^, their use in optical interferometry-based metrological systems has been limited thus far.

**Figure 1:**
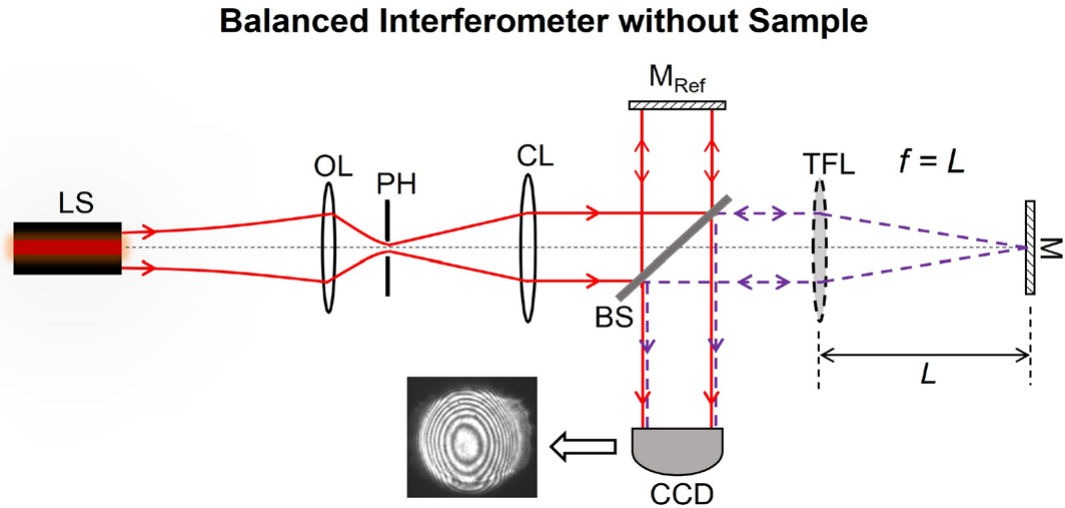
Balanced interferometer without a sample.

**Figure 2:**
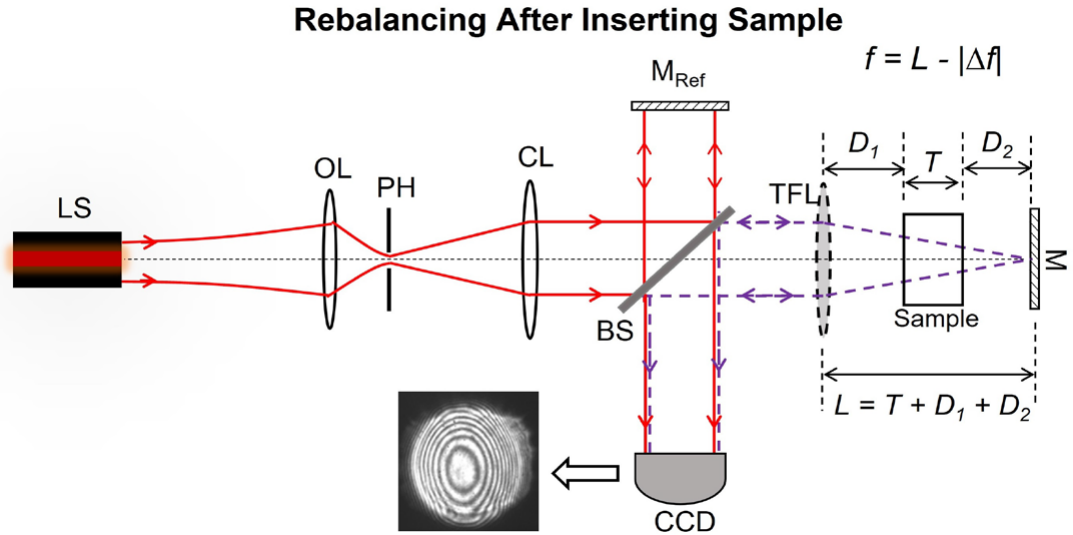
Rebalanced interferometer after sample insertion.

The actively-tunable TG interferometer, which we propose, performs bulk motion-free spatial interferometric measurements without requiring any temporal signal measurements or heterodyne measurements. Instead, the proposed novel electronically-tunable TG interferometer makes measurements by incorporating a TFL—enabling an active beam control within the sample arm of the interferometer. The introduction of the TFL makes this possible as it enables re-balancing of the interferometer without mechanical motion after sample insertion. This proposed actively-controlled TG interferometer setup in its balanced configuration before sample insertion is shown in Fig. 1. Re-balancing the interferometer after sample insertion is simply achieved by retuning the TFL focal length and measuring the change in the focal length required for rebalancing the interferometer allows for characterizing the bulk optical sample thickness/refractive index. This is demonstrated in Fig. 2.

The proposed method of characterizing sample thickness/refractive index can be useful for various applications in optical metrology, material characterization and other measurements. These include, but are certainly not limited to, applications in characterization of optical materials where the refractive index of the material is a desired sample quantity, measuring sample uniformity by scanning the sample and creating a spatial map of the refractive index profile, sample surface quality by creating a spatial map of the sample thickness, quality control applications where random samples are characterized repeatedly, measuring the consistency of partially transmissive liquids in cuvettes or other containers, as well as indirectly measuring the temperature/pressure in closed chambers by measuring the resulting changes in the refractive index inside the chambers. For some advanced applications, the method can be further developed to quantify interferogram distortions after rebalancing and analyzing quality of larger surfaces and checking for surface flatness without needing to scan the surface.

The operation of the proposed actively-tunable is simple, repeatable, bulk motion-free involving only the micro-motion of the liquid inside of the TFL. The resulting bulk motion-free interferometer configuration is a rescalable design which can be altered depending on the target application as well as offering a possibility for potential miniaturization. We characterized multiple samples with the proposed interferometer. These results are presented in the next section and exhibit an excellent agreement with theory.

## Design of the actively tunable Twyman–Green interferometer

### Design and working principle of the actively tunable Twyman–Green interferometer

Neglecting any higher order wavefront aberrations, a classical TG interferometer, shown in Fig. 1 (with a simple spherical lens of a fixed focal length in the place on a TFL), produces bulls-eye fringes on the CCD for any path length difference between the two interferometer arms. Let us assume for simplicity that both interfering beams returning from the sample and reference arms contribute equally at the detection plane, i.e. they have the same electric field amplitude contributions $E_0$ and almost the same $1/e^2$ beam waist radii of *w* at the detection plane. We also assume that the polarization of these two interfering beams are also exactly the same. For the TG interferometer in the unbalanced state, the differential axial propagation phase between the two beams is $\Delta \phi = kz_1 - kz_2$, where $k = 2\pi /\lambda$, $\lambda$ is the quasi-monochromatic optical wavelength, and $z_1$ and $z_2$ are the equivalent optical distances traveled by each respective beam in air before recombining. In this case, the fringe features depend on the difference between the radii of curvature $R_1$ and $R_2$ of the two interfering wavefronts (of $1/e^2$ radii of *w* each) at the CCD plane. The irradiance *I*(*r*) at the CCD plane, with ’*r*’ denoting the radial coordinate in this plane, can be expressed as;

1 $$\begin{aligned}{} & {} I(r) = E_0^2 \exp \left( \frac{-2r^2}{w^2}\right) \left| \exp \left( \! -i \left[ kz_1 + k \frac{r^2}{2R_1} \right] \!\right) + \exp \left[ \! -i \left( kz_2 + k \frac{r^2}{2R_2} \!\right) \right] \right| ^2 \end{aligned}$$

2 $$\begin{aligned}{} & {} I(r) = E_0^2 \exp \left( \frac{-2r^2}{w^2}\right) \left[ 2 + 2\cos \left( \Delta \phi + \frac{kr^2}{2} \left[ \frac{1}{R_1} - \frac{1}{R_2} \right] \right) \right] . \end{aligned}$$

3 $$\begin{aligned}{} & {} I(r) = E_0^2 \exp \left( \frac{-2r^2}{w^2}\right) \left[ 2 + 2\cos \left( \Delta \phi + \frac{kr^2}{2} \left[ \frac{\Delta R}{R_1 R_2} \right] \right) \right] . \end{aligned}$$

Provided that $\Delta \phi = 0$, the fringe pattern on the CCD as a function of the radial coordinate ’*r*’ is given as;

4 $$\begin{aligned} I(r) = E_0^2 \exp \left( \frac{-2r^2}{w^2}\right) \left[ 2 + 2\cos \left( \frac{kr^2}{2} \left[ \frac{\Delta R}{R_1 R_2} \right] \right) \right] . \end{aligned}$$

In the ideal case, for a perfectly balanced interferometer, $R_1 = R_2$ (regardless of the beam collimation quality). In such an ideal scenario, the resulting ideal two-beam interference on the CCD should result in a Gaussian irradiance distribution on the CCD plane (equivalent to one large bright fringe in the CCD plane). Practically, large (yet finitely thick) zero-order fringes are observed as $R_1 \approx R_2$, yet $\Delta R = |R_2 - R_1| \ne 0$ which is described by the expression in Eq. (4).

To measure the thickness or the refractive index using a conventional TG interferometer, the interferometer is balanced before the sample is inserted in the measurement arm. The interferometric balancing is evidenced via the production of thick zero-order fringes on the CCD. The insertion of a transparent sample with parallel sides causes the interferometer to become optically unbalanced. The mirror M is then translated axially to re-balance the interferometer. Rebalancing is verified when the zero-order fringes are reproduced on the CCD while the sample is present in the sample arm. The mirror translation that results in this re-balancing after sample introduction is then used to estimate the thickness or the refractive index of the test sample (provided that only one of those quantities is unknown).

The proposed TFL-based actively-tunable TG interferometer shares the same operation principle as the conventional TG interferometer—but unlike the conventional TG interferometer—rebalancing the interferometer after sample insertion does not involve any motion of bulk optical components. For the proposed configuration, the TG interferometer is first balanced (before sample insertion) by tuning the TFL focal length to a value $f = f_{\textrm{B}}$ (resulting in the thickest possible zero-order fringes on the CCD). This balanced configuration is depicted in Fig. 1. As expected, the interferometer unbalances upon the insertion of the test sample as the minimum beam focal spot shifts and consequently ceases to coincide with the plane of the mirror M any longer.

**Figure 3:**
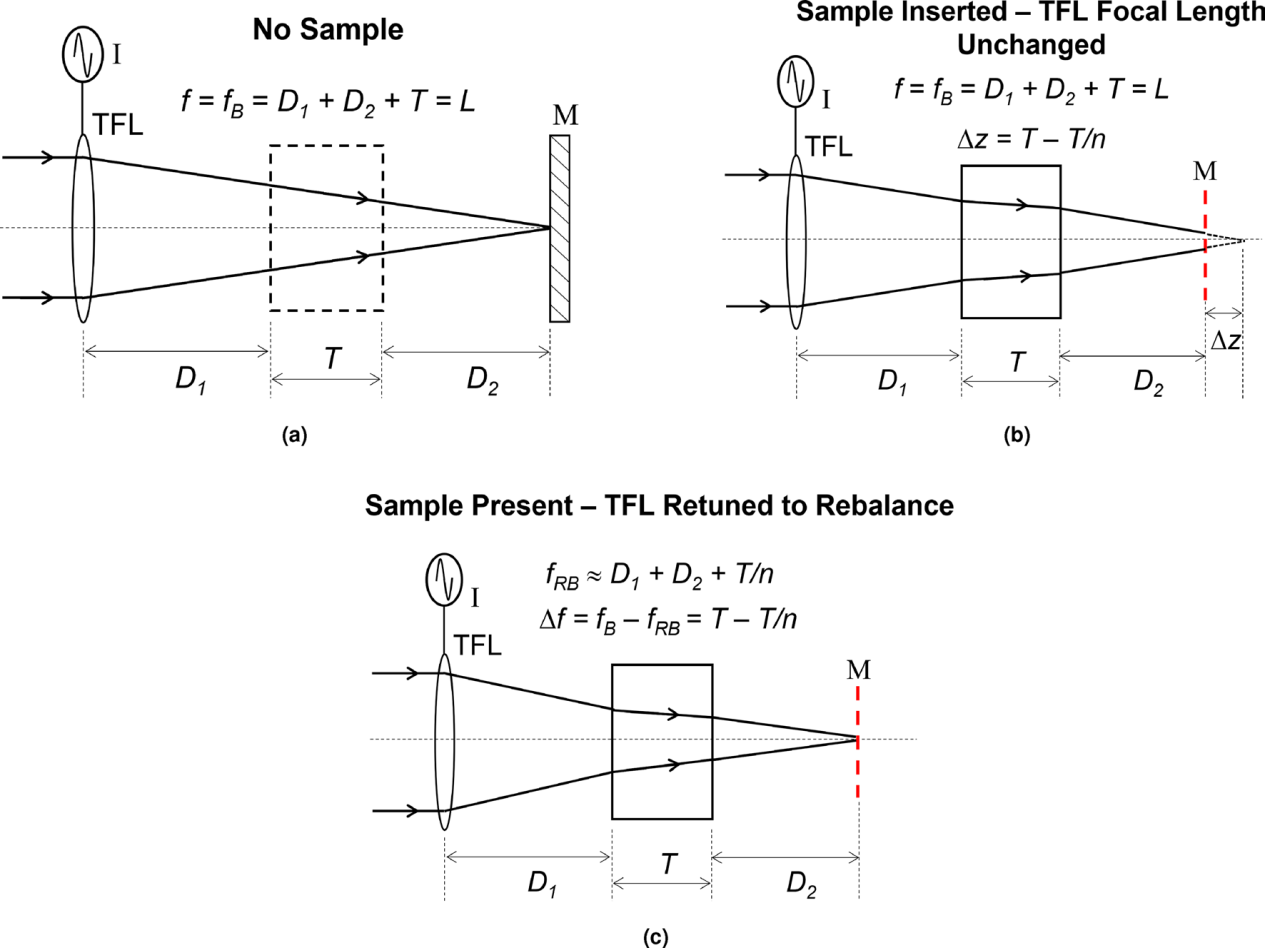
Change in the effective air-equivalent propagation distance $D_\mathrm{eff}$ before and after sample insertion and the required focal length retuning to regain interferometric balance after sample insertion.

Next, the interferometer is rebalanced such that zero-order bulls-eye fringes appear again at the detection plane. Unlike conventional TG interferometers, rebalancing in the proposed TG interferometer is achieved by electronically retuning the TFL focal length to a new value $f_{\textrm{RB}}$ instead of translating the mirror M. When $f = f_{\textrm{RB}}$, zero-order fringes appear at the CCD again which indicates the successful re-balancing if the interferometer and the formation of the minimum beam focal spot at the mirror M plane. By measuring the change in the TFL focal length $\Delta f = |f_{\textrm{B}} - f_{\textrm{RB}}|$ which rebalances the interferometer, we can estimate either the thickness or the refractive index of the test sample—instead of relying on recording the magnitude of bulk mechanical motion to achieve the same. This mode of operation of the proposed interferometer is depicted in Fig.3. The focal length change required for interferometric rebalancing is given by $\Delta f = T - D_\mathrm{eff}$ where $D_\mathrm{eff}$ is the effective air-equivalent distance propagated inside the sample. From standard ABCD matrix formulation^24^, we estimate that

5 $$\begin{aligned} \left[ \begin{matrix} A &{} B \\ C &{} D \end{matrix} \right] = \left[ \begin{matrix} 1 &{} D_\mathrm{eff} \\ 0 &{} 1 \end{matrix} \right] = \left[ \begin{matrix} 1 &{} 0 \\ 0 &{} n \end{matrix} \right] \left[ \begin{matrix} 1 &{} T \\ 0 &{} 1 \end{matrix} \right] \left[ \begin{matrix} 1 &{} 0 \\ 0 &{} 1/n \end{matrix} \right] = \left[ \begin{matrix} 1 &{} T/n \\ 0 &{} 1 \end{matrix} \right] . \end{aligned}$$

Hence, $D_\mathrm{eff} = T/n$, and $\Delta f = T - T/n$. From this we can express $\Delta f$ as;

6 $$\begin{aligned} \Delta f = T\left( 1 - \frac{1}{n} \right) = \left( \frac{n-1}{n} \right) T. \end{aligned}$$

From Eq. (6), the sample thickness *T* or its refractive index *n* can be calculated using the experimentally measured $\Delta f$ as

7 $$\begin{aligned}{} & {} T = \left( \frac{n}{n-1} \right) \Delta f, \end{aligned}$$

8 $$\begin{aligned}{} & {} n = \frac{T}{T - \Delta f}. \end{aligned}$$

Having measured $\Delta f$, sample thickness *T* can be estimated using Eq. (7) if the refractive index *n* is known. Likewise, having measured $\Delta f$, *n* can be estimated from Eq. (8) if *T* is known. We implemented the setup of Fig.2 in the laboratory to demonstrate and verify the operation of the proposed actively-controlled TG interferometer. We measured the thickness and refractive index of multiple samples using our experimental setup. For each sample, we measured $\Delta f$ and used this value to calculate the respective thickness values/refractive indices of these samples from Eqs. (7) and (8). Experiments and results are presented in the following section.

## Experiments and results

### Experimental setup

The actively-tunable TG interferometer of Fig. 2 was implemented with the Optotune EL-16-40 variable focus lens^25^ as the actively tunable focus element (i.e., the TFL). The distance from the TFL to the mirror M was fixed to $L ={280.2}\,{\hbox {mm}}$. A CCD camera was used to capture and record the interferograms. A red He-Ne laser source with a central emission wavelength of $\lambda ={632.8}\,{\hbox {nm}}$ was used as the quasi-monochromatic source. We characterized 5 samples in our experiments to demonstrate and validate the working principle of the proposed actively tunable TG interferometer. In the text, we refer to these samples as ’A’, ’B’, ’C’, ’D’, and ’E’. The interferograms recorded on the CCD were used to estimate $\Delta f$ for each sample. This unique $\Delta f$ value for each unique sample was then used to determine the respective sample *T* or *n*.

### Experimental results

The chosen glass samples A, B, C, and D were transparent with unknown thickness and refractive index values. For each sample, we first measured its refractive index and thickness using standard refractometric measurements and vernier calipers respectively. These measurements serve as baseline *n* and *T* values for each sample and we compare the corresponding *n* and *T* estimates from the tunable TG interferometer with these baseline values for each of the samples A–D. These baseline values are referred to as ’Reference Index’ and ’Reference Thickness’ in Table 1.

We inserted each of the samples within the sample arm of the adaptive TG interferometer and measured $\Delta f$ for each sample by tuning the TFL focal length to $f_{\textrm{RB}}$. These measured $\Delta f$ values are summarized in Table 1.

**Table 1:** Experimental data for different test samples.

Sample	Reference	Reference	$\Delta f$	Estimated	Estimated	Percentage (%)	Percentage (%)
Number	Thickness(mm)	Index	(mm)	Thickness(mm)	Index	Difference in T	Difference in n
A	43.48 $\pm 0.01$	1.482	14.06 $\pm 0.12$	43.24 $\pm 0.24$	1.478 $\pm 0.006$	0.553	0.267
B	43.32 $\pm 0.01$	1.501	14.49 $\pm 0.12$	43.36 $\pm 0.23$	1.503 $\pm 0.007$	0.101	0.051
C	42.40 $\pm 0.01$	1.493	14.22 $\pm 0.12$	43.03 $\pm 0.23$	1.505 $\pm 0.007$	1.476	0.734
D	43.22 $\pm 0.01$	1.493	14.08 $\pm 0.12$	42.62 $\pm 0.23$	1.483 $\pm 0.007$	1.382	0.677
E	25.40 $\pm 0.254$	1.713	10.61 $\pm 0.13$	25.50 $\pm 0.19$	1.717 $\pm 0.015$	0.401	0.287

For each of the samples A–D, the experimentally measured $\Delta f$ value and its ’Reference thickness’ value were used to estimate the respective sample refractive index from Eq. (8). This refractive index estimate for a ’known’ thickness (reference thickness from the vernier calipers) for each sample is listed as ’estimated index’ in Table 1. Similarly, the measured $\Delta f$ value and the ’Reference index’ value (from the refractometric characterization of each sample) for each sample were used to estimate the respective sample thickness value. These thickness estimates for each sample are labeled as ’Estimated thickness’ in Table 1. These estimated sample thickness and index values were then compared to the corresponding reference sample thickness and index values (i.e., the baseline values). The magnitude of the percentage difference between the estimated and reference values for each sample is also presented in Table 1. From Eqs. (20) and (24)—developed and presented in the methods section later—we calculate the errors $\delta T$ and $\delta n$ in the estimates of sample thickness and refractive index respectively. These error estimates are calculated using the minimum thickness measurement ability of the digital vernier $\epsilon _T = 0.01\textrm{mm}$ and minimum applied TFL current step size $[\delta I_\mathrm{DC}]_\mathrm{Min} = 0.07\textrm{mA}$ for the Optotune Lens Driver 4i^26^. These estimation errors are also included to the thickness and refractive index estimates in Table 1.

For glass samples A–D, we observe that the thickness and refractive index values obtained from the tunable TG interferometer closely resemble the corresponding baseline measurements obtained from vernier calipers and refractometric measurements respectively. For these four samples, the thickness estimates obtained from the adaptive TG interferometer remained within $\pm 1.5\%$ of their corresponding baseline values with a mean absolute percentage difference of $0.783\%$. Similarly, the refractive index estimates from the tunable TG interferometer had a mean difference of $0.403\%$ compared to the baseline values for these samples and none of the individual refractive index estimates exceeded $\pm 1\%$.

For samples A-D, commercially measured baseline values were not available. Therefore, sample measurements using the adaptive TG interferometer could only be compared to laboratory measurements with vernier calipers and refractometry. Despite the excellent agreement between thickness and index estimates from the tunable TG interferometer and the corresponding reference baseline values, we wanted to compare the performance of the proposed adaptive TG interferometer to a commercially-characterized sample.

For the purpose of validating and comparing the efficacy of our method with a commercially-measured and toleranced sample, we characterized a 1-inch Thorlabs cm1-pbs251 polarizing beam splitter cube^27^ (sample E), manufactured from an N-SF1 material type of refractive index 1.7125 at a ${633}\,{\hbox {nm}}$ wavelength^28^. This measurement of sample E pitted our proposed method against the sample characterization technique deployed by Thorlabs Inc. The results of sample E measurements are also summarized in Table 1. The thickness and refractive index estimates obtained from the tunable TG interferometer were found to be well within the $\pm 1\%$ tolerance range published by Thorlabs Inc. The accuracy of our results, which are well within the tolerance limits from Thorlabs Inc., validates the accuracy and efficacy of measurements using the proposed actively-tunable TG interferometer.

**Figure 4:**
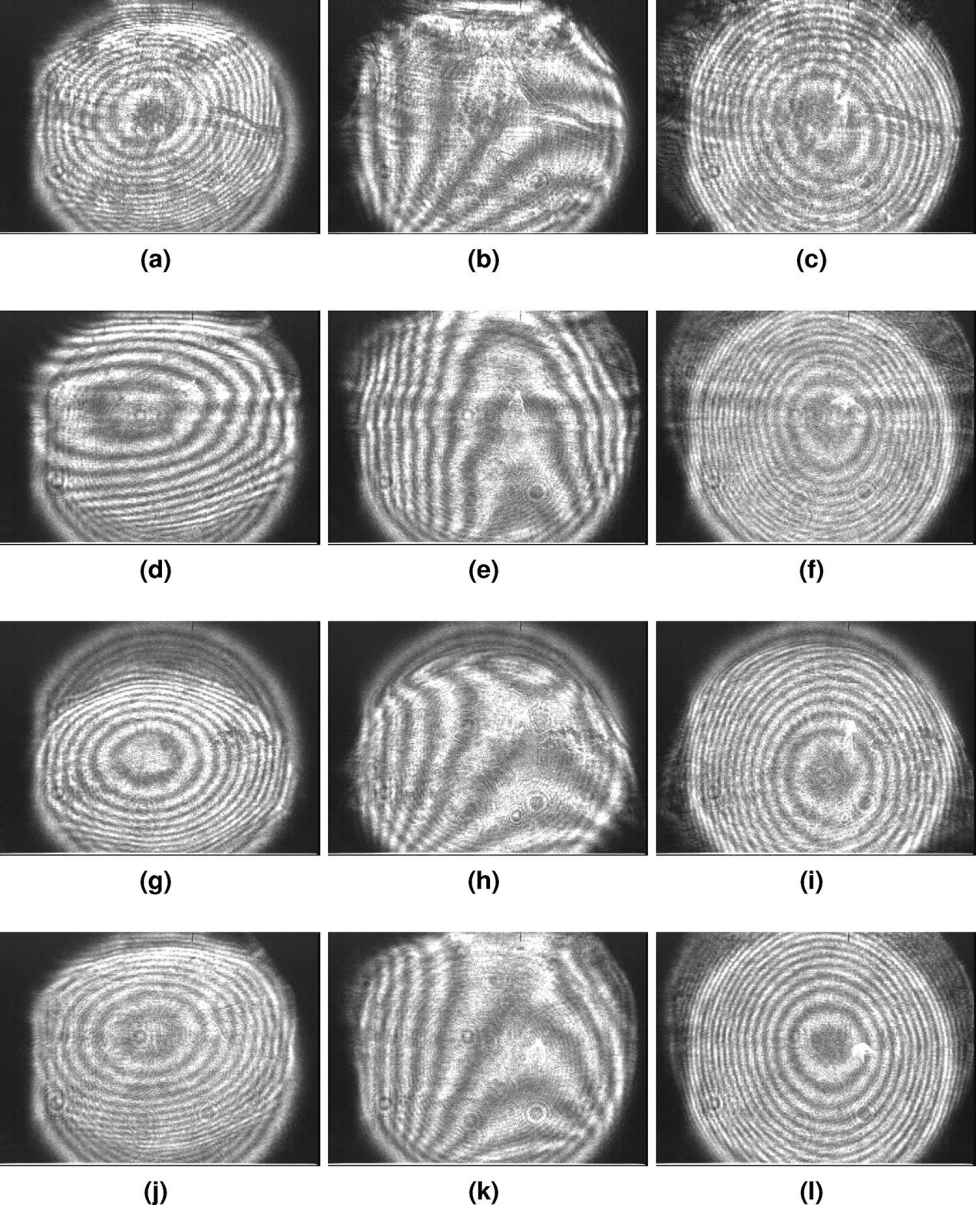
Interferograms recorded by the CCD where image sets (**a**–**c**), (**d**–**f**), (**g**–**i**), and (**j**–**l**) correspond to glass samples A, B, C, and D with each respective 3-image sequence signifying TFL focal length states of $f = f_{\textrm{RB}} -1\textrm{cm}$, $f=f_{\textrm{RB}}$, and $f = f_{\textrm{RB}} +1\textrm{cm}$ i.e., central images (**b**), (**e**), (**h**), and (**k**) are balanced state interferograms for samples A, B, C, and D respectively.

**Figure 5:**
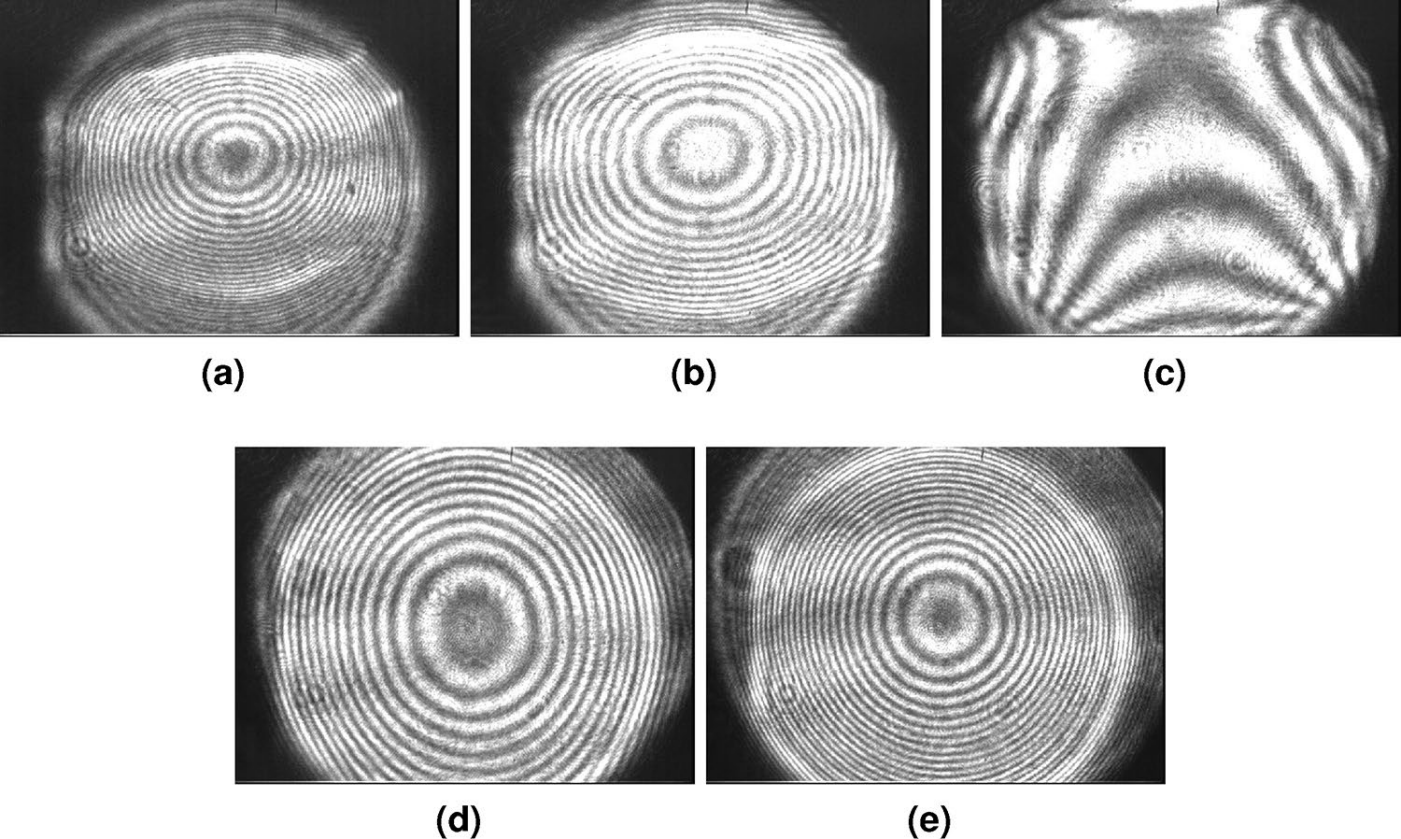
CCD Interferograms for sample ’E’ (Thorlabs beam splitter cube) with TFL focal length tuned from higher to lower values and passing through the balanced interferometer configuration (in Fig.5c). Focal length values correspond to (**a**) $f=222\textrm{mm}$, (**b**) $f=227\textrm{mm}$, (**c**) balanced interferometer interferogram at $f=237\textrm{mm}$, (**d**) $f=247\textrm{mm}$, and (**e**) $f=252\textrm{mm}.$

For each sample, we recorded a sequence of interferograms as captured by the CCD camera after each respective sample was inserted into the interferometer causing it to unbalance. The sequence of images then shows the evolution of the interferogram as the TFL focal length was tuned to the optimal $f_{\textrm{RB}}$ value and beyond. Each picture sequence—one for each sample—comprises three pictures for $f<f_{\textrm{RB}}$, $f=f_{\textrm{RB}}$, and $f>f_{\textrm{RB}}$. This evolution of the interferograms for samples A–D is presented in Fig. 4. Similarly, Fig. 5 shows the evolution of the fringes within the interferogram for sample E characterization. Each sequence shows how non-optimal fringes at $f\ne f_{\textrm{RB}}$ differ from the optimal thickest fringes when $f \approx f_{\textrm{RB}}$. The interferograms depict the presence of astigmatism at best focus^29^ which (along with coma) are typically the dominant wavefront aberrations induced by TFLs^30^. We also include a Supplementary Video showing the evolving interferogram for sample A as the TFL focal length is tuned around the optimal $f_{\textrm{RB}} = 266.3$ mm value for this sample. The video is a more comprehensive depiction (compared to the three-picture sequence for each sample) of the interferogram fringe pattern evolution with a changing TFL focal length.

To ensure repeatability of the process, we ensured that the changes in the interferograms with the tuning of the TFL focal length from $f_{\textrm{B}}$ to $f_{\textrm{RB}}$ were obtained repeatably by tuning the focal lengths bi-directionally over multiple cycles. The process was repeated at least five times for each sample. There was no noticeable change between different TFL tuning cycles. It also has to be noted that any of the conventional image processing methods from prior art, which have been traditionally employed for reading interferograms, can be used for the interferograms obtained with our proposed interferometer. The focus of this paper remains on characterizing samples with our proposed interferometer configuration and not particularly deploying any of the various existing methods of reading interferograms. As our approach required to only determine the zero order fringes, we used a simple fringe thickness estimation via contour extraction to determine the thickness of the thickest fringe allowing us to determine $f_{\textrm{B}}$ and $f_{\textrm{RB}}$. This method entails minor spatial digitization errors due to the use of a pixelated camera, and some possible minor algorithmic errors in contour detection to determine fringe thickness.

Various image processing methods for extracting features from interferograms have been proposed in prior art. For example, Malacara et al.^31^ and Maciel et al.^32^ describe various signal processing techniques for reading and interpreting interferograms. Some more recent interferogram reading techniques are based on machine learning-based approaches such as^33^. For our analysis, we use a simple and direct method. We begin by capturing the interferogram images, thresholding the images to convert these images from gray-scale to black-and-white, and determining the center of the interferogram ($x_0, y_0$) by means of a simple center of mass algorithm. Then we manually measured the intensity line profiles along difference linear directions (lines) which passed through ($x_0, y_0$). The largest intensity plateau in any of the line profiles was taken to be the largest dimension of the largest fringe. The process of reading fringes and determining the dimension of the largest fringe could be further refined by automating the step of measuring line profiles as well as deploying more advanced image processing algorithms to read the dimensions of the largest fringe in an interferogram.

## Discussion on the operation of the proposed Twyman–Green interferometer

### Some advantages and limitations of the proposed tunable TG interferometer configuration

As is apparent from the tunable TG interferometer configuration, we characterize samples by inserting them into the path of a converging beam arriving from the TFL and a diverging beam on the path back from the mirror M. This measurement is different from Michelson interferometer measurements where samples are inserted in the path of a collimated beam or Twyman–Green interferometer measurements^10^—where gradual changes in the fringe pattern or visibility is measured by means of incurring gradual changes to the samples under test. Placing the sample in the path of a non-collimated beam overcomes the limitation of keeping a track of changes in the fringe patterns as a result of necessary gradual changes which must be made in the sample arm of the interferometer. The abrupt change in the optical path length can be measured with the proposed interferometer because the tunable TG interferometer does not rely on constantly keeping a track of changes to the fringes recorded at the detector.

Placing the sample in the path of a non-collimated beam does come with a few drawbacks. A converging or diverging beam probing a samples results in a different incident beam size at the two interfaces of the sample. This results in the interface at which the beam size is larger to produce more distortions to the recorded interferogram than the surface with a smaller incident beam. Ideally, it is desirable that both sample interfaces contribute equally to interferogram distortions as a result of surface imperfections but this is not the case with our proposed configuration. This effect does not affect simple thickness or refractive index measurements greatly but it would if we are using the same setup for surface quality measurements.

Sometimes a more comprehensive sample characterization may require the sample to be translated across the normally incident beam on it inside the sample arm. This kind of measurement can provide a spatial map of a sample thickness or refractive index. With the sample placed in the path of a non-collimated beam, the two sample interfaces are probed with two different beam sizes i.e. two different spatial sample sizes. Although this is not the case with our measurements, but the proposed configuration will incur this potential drawback if it is put to use in a scanning sample configuration.

The placement of a sample in the path of a converging beam allows accurate thickness and refractive index measurements for samples with a uniform thickness/refractive index. Measurement of non-uniform refractive index samples’ profiles, while interesting, is outside the scope of this current study and may be tackled separately in future work.”

### Effects of thermal stability of the TFL on the measurements

**Figure 6:**
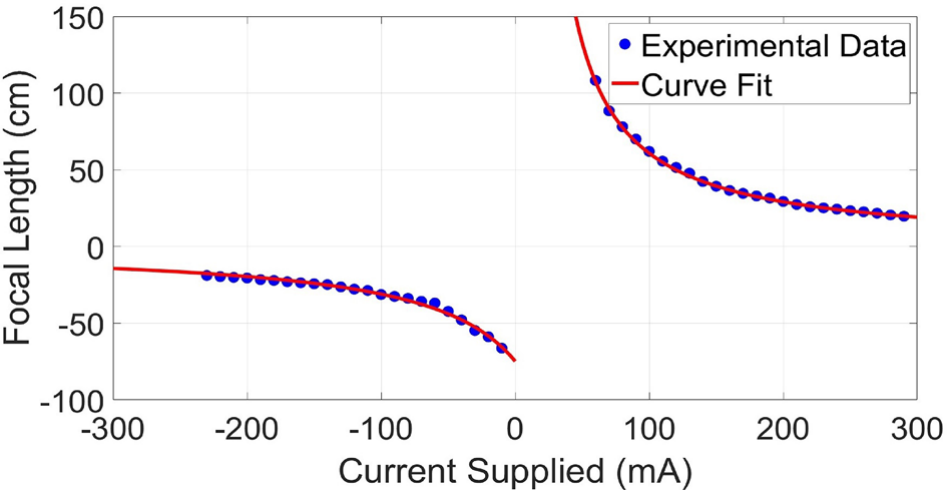
The Optotune EL-16-40 TFL focal length as a function of the applied current as characterized in the lab and approximated by best fit focal length as a function of the applied current $I_\mathrm{DC}$ to the TFL.

The thermal stability and performance of Optotune TFLs was studied recently by Marrakchi et al. ^34^. It was demonstrated that the TFL temperature rises as it is kept on for extended periods of time affecting the effective optical power of the TFL and causing it to change over time significantly. The shift in the optical power with time effectively results in shifting the focal length plots up or down in Fig. 6. The thermal state of the lens and the resulting shift in focal length would affect our measurements significantly if we depended on measuring a single absolute focal length value to determine sample thickness or refractive index.

In our case though, we calculate the difference between the TFL focal lengths in the balanced and rebalanced states. A shift in this difference would be minimal if $f_{\textrm{B}}$ and $f_{\textrm{RB}}$ are measured within a short space of time at almost the same thermal state of the TFL before and after sample insertion. There will be some minimal residual errors as a result of any inherent thermal instabilities of the TFL. Another thing to note here is that for most practical samples $f_{\textrm{B}}$ and $f_{\textrm{RB}}$ are reasonably close compared to the entire focal length tuning range of the lens. This mitigates any errors due to possibly atypical focal length shifts close to the minimum and maximum focal lengths of the TFL operation.

The TFL can be characterized and calibrated for different temperature conditions depending on the operating temperature of the application(s) for which the proposed system is to be deployed. A relevant TFL recalibration enables thickness/refractive measurements at a different temperature.

## Methods

### Focal length characterization of the TFL used for the experiments

We characterized the focal length of the TFL as a function of the applied DC current $I_\mathrm{DC}$ from the Optotune lens driver model 4i. For this purpose, we used a simple setup where a collimated beam was passed through the TFL. The location of the minimum focal spot was recorded for different input current $I_\mathrm{DC}$ values to the TFL. The distance of the minimum focal spot from the TFL equalled the TFL focal length at each corresponding applied $I_\mathrm{DC}$. For each applied $I_\mathrm{DC}$, a CCD, mounted on an axial translation stage, was translated along the axial beam propagation direction to determine the corresponding location of minimum beam focus. The measurements were repeated for multiple TFL input current values. The measured focal length, is plotted as a function of the input current in Fig. 6. We also plot the best fit functions of type $f = A/\left( I_\mathrm{DC} + B \right)$ to obtain;

9 $$\begin{aligned} f = \frac{5608}{I_\mathrm{DC} -7.801} ~~~ \textrm{for} ~~ f > 0, \end{aligned}$$

and

10 $$\begin{aligned} f = \frac{5297}{I_\mathrm{DC} -70.83} ~~~ \textrm{for} ~~ f < 0. \end{aligned}$$

The fitting functions assume focal length values in centimeters and the applied current $I_\mathrm{DC}$ to the TFL in milliamperes.

### Measurement error estimates

An estimate for the measurement error in the estimates for ’*T*’ or ’*n*’ can be determined from Eqs. (7) and (8) respectively. Here, we derive expressions to estimate the approximate errors in the thickness and refractive index measurements from the tunable TG interferometer assembly.

#### Error in thickness measurements

The error $\delta T$ in the estimate of the sample thickness can be calculated as

11 $$\begin{aligned} \delta T = \sqrt{\left( \frac{\partial T}{\partial n} \varepsilon _\mathrm{n}\right) ^2 + \left( \frac{\partial T}{\partial \Delta f} \varepsilon _\mathrm {\Delta f}\right) ^2 }, \end{aligned}$$

where the $\varepsilon _\mathrm{n}$ represents the error in the baseline value of ’*n*’ and $\varepsilon _\mathrm {\Delta f}$ is the uncertainty in the focal length change $\Delta f$ which is required to rebalance the interferometer after sample insertion. The uncertainty in the focal length step size $\varepsilon _\mathrm {\Delta f}$ is the minimum focal length step size which the Optotune lens driver 4i enables. We estimate $\varepsilon _\mathrm {\Delta f}$ from Eq. (9) where the focal length step size depends on the minimum applied current step size $\left[ \delta I_\mathrm{DC} \right] _\mathrm{Min}$ and the uncertainties $\Delta A$ and $\Delta B$ in the curve-fitting coefficients ’A’ and ’B’. It has to be noted that units for *T*, $\Delta f$, and $\varepsilon _\mathrm {\Delta f}$ must all be chosen as same. The dependence of $\varepsilon _\mathrm {\Delta f}$ on $\left[ \delta I_\mathrm{DC} \right] _\mathrm{Min}$, $\Delta A$, $\Delta B$, and any other system calibration errors $\Delta _\mathrm{Cal}$ such as errors, due to spatial quantization etc, is given as

12 $$\begin{aligned} \varepsilon _\mathrm {\Delta f} = \sqrt{\left( \frac{d f}{d I_\mathrm{DC}}\left[ \delta I_\mathrm{DC} \right] _\mathrm{Min} \right) ^2 + \left[ \Delta A \right] _\mathrm{Est}^2 + \left[ \Delta B \right] _\mathrm{Est}^2 + \left[ \Delta _\mathrm{Cal} \right] ^2} \end{aligned}$$

where $\left[ \Delta A \right] _\mathrm{Est} \approx (df/dA)(\Delta A)$, and $\left[ \Delta B \right] _\mathrm{Est} \approx (df/dB) (\Delta B)$. With $f = A/\left( I_\mathrm{DC} + B \right)$ for positive lens operation,

13 $$\begin{aligned}{} & {} \frac{d f}{d I_\mathrm{DC}} = -\frac{A}{\left( I_\mathrm{DC} + B \right) ^2}, \end{aligned}$$

14 $$\begin{aligned}{} & {} \frac{d f}{d A} = \frac{1}{\left( I_\mathrm{DC} + B \right) }, \end{aligned}$$

and

15 $$\begin{aligned} \frac{d f}{d B} = -\frac{A}{\left( I_\mathrm{DC} + B \right) ^2}. \end{aligned}$$

This results in

16 $$\begin{aligned} \varepsilon _\mathrm {\Delta f} = \sqrt{\left( - \frac{A}{\left( I_\mathrm{DC} + B \right) ^2} \left[ \delta I_\mathrm{DC} \right] _\mathrm{Min}\right) ^2 + \left( \frac{\Delta A}{\left( I_\mathrm{DC} + B \right) } \right) ^2 + \left( - \frac{A}{\left( I_\mathrm{DC} + B \right) ^2} \Delta B \right) ^2}. \end{aligned}$$

From Eq. (7)

17 $$\begin{aligned} T = \left( \frac{n}{n-1} \right) \Delta f, \end{aligned}$$

and

18 $$\begin{aligned} \frac{\partial T}{\partial \Delta f} \approx \frac{n}{n-1}. \end{aligned}$$

If we ignore the uncertainty $\varepsilon _\mathrm{n}$ in the refractive index estimates—partly because of unavailability of uncertainty data on the he Schott glass catalog—we obtain

19 $$\begin{aligned} \delta T \approx \pm \frac{\partial T}{\partial \Delta f} \varepsilon _\mathrm { \Delta f}. \end{aligned}$$

Substituting $\varepsilon _\mathrm {\Delta f}$ and $\partial T / \partial \Delta f$ from Eqs. (16) and (18) into Eq. (19), we obtain the error $\delta T$ in the estimate of sample thickness in terms of the known refractive index, the operating input current $I_\mathrm{DC}$, and the minimum TFL focal length step size $\left[ \delta I_\mathrm{DC} \right] _\mathrm{Min}$. This is given as

20 $$\begin{aligned} \delta T = \left( \frac{n}{n-1} \right) \sqrt{\left( - \frac{A}{\left( I_\mathrm{DC} + B \right) ^2} \left[ \delta I_\mathrm{DC} \right] _\mathrm{Min}\right) ^2 + \left( \frac{\Delta A}{\left( I_\mathrm{DC} + B \right) } \right) ^2 + \left( - \frac{A}{\left( I_\mathrm{DC} + B \right) ^2} \Delta B \right) ^2 + [\Delta _\mathrm{Cal}]^2}. \end{aligned}$$

With our fitting function plotted in Fig. 6 for the positive focal length operation of the TFL operation, our fitting coefficients are $A = 56080 \pm 5.8$ and $B = -7.801 \pm 0.09$ (for focal length in millimeters). To minimize uncertainty in the thickness estimate due to uncertainty in fitting coefficients, we can use different fitting functions such as higher-order polynomials to model the focal length curve of the TFL to a greater accuracy. We can also use piece-wise curve-fitting for better analytical approximations.

#### Error in refractive index measurements

Similar to how we estimated error in the thickness measurements, we determine the error in the sample refractive index estimate that the proposed TG interferometer yields. The error $\delta n$ in the refractive index estimate can be calculated as

21 $$\begin{aligned} \delta n = \sqrt{\left( \frac{\partial n}{\partial T} \epsilon _\mathrm{T}\right) ^2 + \left( \frac{\partial n}{\partial \Delta f} \epsilon _\mathrm {\Delta f}\right) ^2 }, \end{aligned}$$

where the $\varepsilon _\mathrm{T}$ represents the error in the baseline value of ’*T*’ and $\varepsilon _\mathrm {\Delta f}$, as earlier, represents the uncertainty in $\Delta f$. From Eq. (8), obtain

22 $$\begin{aligned} \frac{\partial n}{\partial T} = -\frac{\Delta f}{\left( T - \Delta f \right) ^2 }, \end{aligned}$$

and

23 $$\begin{aligned} \frac{\partial n}{\partial \Delta f} = \frac{T}{\left( T - \Delta f \right) ^2}. \end{aligned}$$

Therefore,

24 $$\begin{aligned} \delta n = \frac{1}{\left( T - \Delta f \right) ^2} \sqrt{ \left( [\Delta f] [\varepsilon _\mathrm{T}]\right) ^2 + \left( [T][\varepsilon _\mathrm {\Delta f}]\right) ^2 }, \end{aligned}$$

where $\varepsilon _\mathrm{T}$ is the error in the measurement of the baseline thickness value. In our case, it is the instrument error of the vernier calipers used for the thickness measurements. On the other hand, $\varepsilon _\mathrm {\Delta f}$ is determined from Eq. (16) as a function of the minimum current step size $\left[ \delta I_\mathrm{DC} \right] _\mathrm{Min}$.

### Focal length step size

The minimum focal length step size $\delta f$ of the TFL depends on the minimum step size of the applied current $I_\mathrm{DC}$ and the regime of focal length range over which the TFL is being operated. Disregarding any errors from uncertainties in the fitting coefficients (as the actual focal length step size does not depend on the mathematical fitting function that one uses to analytically model TFL behavior), this can be directly calculated from Eqs. (12), (13), and (16) as

25 $$\begin{aligned} \delta f = \pm \frac{A}{\left( I_\mathrm{DC} + B \right) ^2} \left[ \delta I_\mathrm{DC} \right] _\mathrm{Min}. \end{aligned}$$

For the focal length range of operation for our experiments and with a minimum current step size $\left[ \delta I_\mathrm{DC} \right] _\mathrm{Min}$ of $0.07\textrm{mA}$, we obtain an average minimum focal length step size of $\bar{\delta f} \approx \pm 0.075\textrm{mm}$.

## Conclusion

In this paper, we have presented an electronically tunable Twyman–Green interferometer configuration which can be used to characterize the thickness or the refractive index of parallel plate transparent samples involving no bulk mechanical motion of optical elements. The bulk motion-free operation of the interferometer is achieved via the deployment of a tunable focus lens (TFL) in the sample arm of the TG interferometer. We show that the interferometric unbalancing caused by sample insertion is countered via TFL focal length retuning. This magnitude of focal length retuning for interferometric rebalancing can be used to determine sample properties. We also validated the proposed tunable TG interferometer configuration and demonstrate its working principle with carefully designed experiments where we implemented the interferometer and characterized multiple samples and compared the interferometric measurements to baseline thickness and refractive index values obtained using vernier calipers and from the Schott optical glass catalogue respectively. For most samples, the measurements from the interferometric measurements mostly fell within a $\pm 1\%$ error compared to baseline values. Some measured values were between $1\%$ and $1.5\%$ compared to their respective baseline values.

## Supplementary Information

Supplementary Legend.

Supplementary Video 1.

## Data Availability

All data generated or analysed during this study are included in this published article and its supplementary information files.
