# Supplementary material for: Robust, motion-free optical characterization of samples using actively-tunable Twyman–Green interferometry
Source: Sci Rep. 2023 Apr 7;13:5678. doi: 10.1038/s41598-023-32791-2 (PMC10082213; doi:10.1038/s41598-023-32791-2)
Supplement: Supplementary file 1 — Supplementary Legend. [file 41598_2023_32791_MOESM1_ESM.docx]

**Supplementary Video S1**

The video depicts tuning the focal length of the TFL by starting with a focal length which is less than the interferometer rebalancing focal length, then arriving at the rebalancing focal length and then going beyond it. The zero-order fringes are observed when the TFL focal length values equals the required interferometer rebalancing focal length.
